# Comparison of blood gas parameters, ions, and glucose concentration in polish Holstein-Friesian Dairy cows at different milk production levels

**DOI:** 10.1038/s41598-023-28644-7

**Published:** 2023-01-25

**Authors:** Bartosz Pawliński, Marcin Gołębiewski, Michał Trela, Olga Witkowska-Piłaszewicz

**Affiliations:** 1 Department of Large Animals Diseases and Clinic, Institute of Veterinary Medicine, Warsaw University of Life Sciences, Nowoursynowska 166, 02-787 Warsaw, Poland; 2 Department of Animal Breeding, Institute of Animal Sciences, Warsaw University of Life Sciences, Nowoursynowska 166, 02-787 Warsaw, Poland

**Keywords:** Zoology, Biomarkers, Diseases, Health care

## Abstract

Genetic selection for increased milk yield has been a key driver of dairy intensification. The modern dairy cow produces much higher amounts of milk than the cattle of several years ago, and this may have an influence on hematologic values at different stages of lactation and on cows with different levels of milk production. The purpose of the study was to investigate the variations in blood parameters such as Ht, tHb, sO_2_, FO_2_Hb, FCOHb, FMetHb, FHHb, pH, pCO_2_, pO_2_, standard HCO_3_−, actual HCO_3_−, BE, BE ecf, ctCO_2_, BO_2_, p50, and ctO_2_ in cows at different milk production levels. In addition, ions such as Na+, K+ , Ca++, Ca++ (7.4), and Cl−, and AnGap and glucose were examined. Our findings indicated that differences in the examined blood parameters between low and high-production dairy cattle do exist. The most apparent differences were connected with blood pH (*p* < 0.01), oxygen metabolism (Ht, tHb, sO2, FO2Hb; *p* < 0.01), and glucose utilization (*p* < 0.01) The results confirm that the parameters connected with blood oxygen metabolism and glucose metabolism increase significantly in high-production animals. In conclusion, reference values should be considered in light of the lactation stage and level of milk production, because these might influence how changes should be interpreted. The main limitation of the study is the delay to analysis. However, the blood was properly stored (4C), thus changes were delayed. Anyway, it is very hard in the field practice to perform it within 5 min after the blood collection and according to studies it has low impact on clinical outcomes.

## Introduction

According to a EUROSTAT analysis, the European dairy cattle population consists of approximately 87 million cows, which are kept on about 3.6 million farms. One of the largest dairy producers in the European Union (EU) is Poland, which is responsible for 1.7% of global milk production (14.1 billion liters) and 8.5% of total EU milk deliveries, coming just after the Netherlands (14.6 billion liters, 9.7% of EU milk deliveries in 2019)^1^. Increases in milk production are accompanied by a tendency to diminish the total number of dairy cows and to reduce the size of herds, as well as to create less new, small and mid-size herds^2^. In Poland, during 2019, there was a 2.3% decrease in the dairy cow population in comparison to 2018, whereas milk production increased.

Milk production efficiency is determined by biological and technological progress. According to the Polish Federation of Cattle Breeders and Dairy Farmers the main dairy breed in Poland is the Holstein-Friesian (HF), which makes up approximately 85% of the total number of recorded cows. There is a growing interest in animals with high genetic value that provide high milk production. HF are genetically adapted to ensure theoretically high production rates, thus, breed traits are manifested not only in unique appearances and high-production ability, but also involve differences in some physiological processes. Changes in hematological parameters are highly correlated with changes in milk production^3–5^. The parameters can be used to reflect the health status of the herd in bovine medicine^6,7^. It has been postulated that the evaluation of blood markers may be used as a diagnostic tool to help identify some subclinical disorders such as reproductive disorders^8^ and metabolic disturbances^9,10^, but may also indicate a possible response to some adverse environmental factors^7^. So, a response to external factors may be expressed in the levels of different blood parameters^11^. Blood is a tissue that reflects the health status of the whole organism; thus, blood analysis can be a useful means of detecting the early onset of a pathology.

Hematological reference values may vary due to geographic location, biological rhythms, season, nutrition, age, ambient temperature, and relative humidity and temperature-humidity indexes^12–15^. It has been suggested that the most appropriate reference range is for a group of healthy animals in an environment that is as close to the target patient as possible^16^. In addition, physiological conditions should be closely characterized for each specific group of animals. The reference values were established 10 or more years ago^17,18^, thus, the lower levels of milk production from cows that were taken into consideration were also associated with that time (approx. 18–20 kg milk/cow)^19,20^. In the global literature, there is lack of recently reported values for blood gas parameters for modern dairy cows; most publications are from the 1980–90 s and/or only evaluated a limited number of parameters^15,21–24^. In addition, milk production per cow has increased during this time (from 2009 to 2013 it increased by + 7.5%) [EUROSTAT]. Thus, the aim of this study was to investigate the differences in blood gas parameters and ion concentration in dairy HF cows at different levels of production ability, while also comparing them with commonly accepted reference values for bovine species.

## Methods

Sampling protocols were part of standard veterinary diagnostic procedures and were performed according to European directive EU/2010/63 and Polish legal regulations (*Act of 15 January 2015 on the Protection of Animals Used for Scientific or Educational Purposes*); the approval of the Local Commission for Ethics in Animal Experiments was not required.

### Animals

The study was conducted at an experimental dairy farm belonging to the Warsaw University of Life Sciences (WULS), on a herd of approximately 350 cows that were maintained in a free-stall housing system.

A total number of 112 animals, weighing 550–650 kg each, with a body condition score (BCS) of 3.0–3.5, and averaging 31.9 kg milk/cow/day were selected for the study. The cows were milked twice a day in a herringbone milking parlor at 5 am to 6 am, and 5 pm to 6 pm. The cows were 2–8 years old and were thus during their first and fifth lactations, having between 60 and 125 days of lactation. The inclusion criteria was that the cow was clinically healthy and milk yield production level. Then, based on their mean daily milk yield, the cows were divided into five groups: I—44–39 kg milk/cow (n = 36); II—38–31 kg milk/cow (n = 21); III—30–26 kg milk/cow (n = 27); IV—25–20 kg milk/cow (n = 18); and V—19–17 kg milk/cow (n = 10) (Table 1). There were no symptoms found indicating any disorders during the clinical examination, which included rectal temperature, heart and respiratory rate, mucous membranes (color and moisture), dehydration (measured as the time it takes a section of pinched skin-fold from the point of the shoulder to flatten), gut sounds, rectal examination together with an ultrasound of the reproductive tract, and milk examination using the California Mastitis Test (CMT), as well as monitoring the stable milk production during the last two weeks. The animals were kept in a free-stall system, fed ad libitum with a complete formula (total mixed ration—TMR). The cows’ diet was formulated using a system developed by the French National Institute for Agricultural Research (INRA) to provide adequate milk energy and milk production for a mean live weight of 628 ± 34 kg. During the lactation period, the TMR was adjusted according to the milk performance and the lactation stage. The ingredient composition of the TMR diets, and the balance of nutrient supply between the animals observed in the experiment, is presented in Table 2.

**Table 1:** Cows involved in the study.

Group	n	Mean daily milk yield [kg milk/cow]
I	36	44–39
II	21	38–31
III	27	30–26
IV	18	25–20
V	10	19–17

**Table 2:** The ingredient composition of the total mixed ration (TMR) and nutrient balance for cows at different stages of lactation.

Items	TMR (1)	TMR (2)	TMR (3)	TMR (4)	TMR (5)
*Ingredient (kg/d)*					
Maize silage	27.2	26.3	25.5	24.5	24.1
Alfalfa silage	11.3	11.3	11.3	9.5	9.6
Corn silage	5.0	4.5	4.0	3.7	3.3
Soybean meal	2.1	01.5	01.4	1.2	1.0
Pasture ground chalk	0.1	0.1	0.1	0.1	0.1
VIT-RA BML-vitamin mix2)	0.16	0.16	0.16	0.16	0.16
Salt	0.05	0.05	0.05	0.05	0.05
Rapeseed meal	2.5	2.5	2.5	2.0	2.1
Magnesium oxide	0.05	0.05	0.05	0.05	0.06
*Nutrient supply*					
NEL (Mcal/d)	41.2	39.2	32.1	28.3	26.4
Metabolic protein (g/d)	2.675	2.175	1.999	1.778	1.679
Ca (g/d)	66	62	58	51	50
P (g/d)	58	57	45	32	32
K (g/d)	250	230	210	185	165
Balance (%)					
NEL (Mcal/d)	2.49	4.13	1.89	1.65	1.66
Metabolic protein (g/d)	0.75	3.21	0.55	4.60	4.61
Ca (g/d)	1.89	1.52	1.25	1.89	1.90
P (g/d)	− 2.22	− 1.72	− 0.98	− 3.13	− 3.14
K (g/d)	9.52	8.80	7.24	8.48	8.49

The cows were fed a basal diet (TMR) ad libitum, and the TMR was formulated using a system developed by the French National Institute for Agricultural Research (INRA) to provide adequate milk energy and milk production for cows of 628 ± 34 kg.

*TMR* Total mixed ration, *NEL* Net energy for lactation. 1) TMR 1, group I, 38 kg/day; TMR 2, group II, 44 kg/day; TMR 3, group III, 30 kg/day; TMR 4, group IV, 25 kg/day; TMR 5, group V, 19 kg/day. 2) Vitamin premix; VIT-RA BML (values per kg): 150 g Ca; 100 g P; 50 g Na; 40 g Mg; 9,000 mg Zn; 7,000 mg Mn; 1,000 mg Cu; 100 mg J; 50 mg Se; 1,200,000 IU vitamin A; 120,000 IU vitamin D3; 5,000 mg vitamin E; 93 mg vitamin K; 80 mg vitamin B1; 160 mg vitamin B6; 110 mg vitamin B2; 1,000 μg vitamin B12 (PPH VITRA, Kusowo, Poland).

The animals were under veterinary care every day during the whole period of the experiment. A particular effort was made to exclude any animals with lung and breathing disorders, kidney problems, or an acid-base imbalance by carrying out a physical and, if necessary, a blood examination, as these may have had a significant effect on the blood gas analysis.

### Blood sampling

The blood samples were collected in the morning, directly into 1 mL gasometric syringes (calcium-balanced lithium heparin, The Blood Gas Monovette® 1 ml Sarstedt, Nümbrecht, Germany) by manual aspiration. We decided to use this device to avoid hemolysis influencing the obtained results^25^. The air was removed by attaching a ventilation device and expelling the air from the syringe. The blood was stored at 4 °C within 0.5 h of collection. The oximetric parameters were assessed in whole blood using a RAPIDPoint 500 analyzer (Siemens, Erlangen, Germany).

The following parameters were obtained: tHb—total hemoglobin concentration, Ht—hematocrit, pCO_2_—partial pressure of carbon dioxide, pO_2_—partial pressure of oxygen, HCO_3_ − act—actual bicarbonate concentration, HCO_3_ − std—standard bicarbonate concentration, BE(B)—base excess, BE ecf—base excess of extracellular fluid, ctCO_2_—total carbon dioxide serum concentration, sO_2_—saturation, FO2Hb—oxyhemoglobin, FCOHb—carboxyhemoglobin, FMetHb—methemoglobin, FHHb—deoxyhemoglobin, BO_2_—blood oxygen, p50—hemoglobin-oxygen affinity, and ctO_2_—total oxygen concentration. The HCO_3_ − act, HCO_3_ − std, BE, BE ecf, p50, and sO_2_ were calculated automatically by the blood gas analyzer. In addition, the examination included an analysis of serum electrolytes such as sodium [Na+], potassium [K+], total calcium [total Ca++], adjusted ionized calcium (at pH 7.4) [Ca++ 7.4], chloride [Cl−], and the anion gap [AnGap] and glucose (Glu) concentration.

Measurements were carried out as recommended by the National Committee of Blood Laboratory Standards (*Considerations in the Simultaneous Measurement of Blood Gases, Electrolytes and Related Analytes in Whole Blood; Proposed Guideline*^26^). For each test, the analyzer’s operating temperature was set according to the bovine rectal temperature recorded during sampling.

### Statistical analysis

Statistical analysis was performed in PQStat 1.6.4.121. (Poznan, Poland). The numerical variables were given as the arithmetic median and standard deviation (SD), or as the interquartile range (IQR), unless the variable was normally distributed according to the Shapiro-Wilk W test. Range was presented in all cases. Between-group comparisons were performed using the Kruskal-Wallis H test along with Dunn’s post-hoc test, unless the variable was normally distributed. Additionally, a Jonckheere-Terpstra trend test was performed. The significance level was set at 0.05.

### Ethics approval and consent to participate

All samplings were part of standard veterinary diagnostic procedures, and done according to the EU Directive 2010/63/EU for animal experiments and Polish legal regulations; approval of the Local Commission for Ethics in Animal Experiments was not required.

## Results

The average production performance of the cows exceeded 11,250 kg of milk per lactation, with a 3.42% protein and 4.38% fat content.

**Figure 1:**
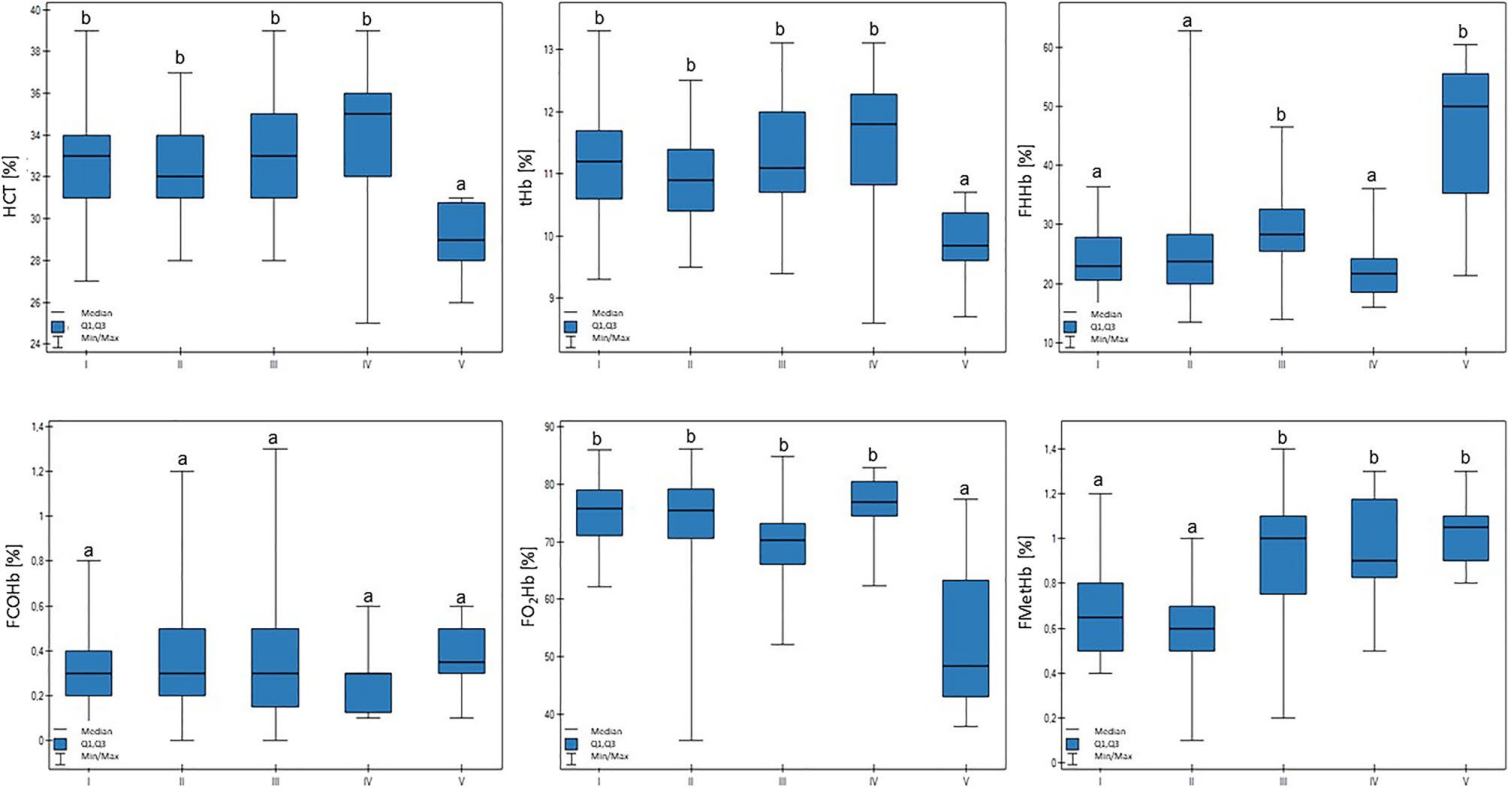
Differences in tHb (total hemoglobin concentration), HCT (hematocrit), FHHb (deoxyhemoglobin), FCOHb (carboxyhemoglobin), FO_2_Hb (oxyhemoglobin), and FMetHb (methemoglobin) values in cows at different production levels. In the box plots, the upper whisker represents the maximum value; the upper line of the box represents Q3 (upper quartile); the center line inside the box represents the median; the lower line of the box represents Q1 (lower quartile); and the lower whisker represents the minimum value. Groups marked by letters (a–b) differ significantly, p ≤ 0.05. The cows were divided in to five groups: I—44–39 kg milk/cow; II—38–31 kg milk/cow; III—30–26 kg milk/cow; IV—25–20 kg milk/cow; and V—19–17 kg milk/cow, based on mean daily milk yield.

The values for the examined blood gas parameters in the cattle from the different groups are presented in Tables 3 and 4. Ht and tHb in group V were lower in comparison to the other groups (*p* < 0.01). The FO_2_Hb value in group V was also lower (*p* < 0.01). FMetHb values differed across the examined groups (*p* < 0.01): the results for groups I and II were lower in comparison to groups III, IV, and V. In addition, there was a trend that confirmed that lower milk production was connected with higher FMetHb values (*p* < 0.1). Group V was characterized by an increase in FHHb compared to the other groups, except for group III (*p* < 0.01). These results are presented in Fig. 1.

**Table 3:** Blood gasometrical parameters (median, SD, minimal, and maximal values) in different groups of dairy cattle compared with the normal values for cattle (according to Sayers et al. 2016^31^, Wood et al. 2010^40^, Temiz et al. 2014^37^, Indrova et al. 2020^20^, Cornell Animal Health Diagnostic Center^42^).

Parameter	Group I (n = 36)	Group II (n = 21)	Group III (n = 27)	Group IV (n = 18)	Group V (n = 10)	Normal values for bovine species
pH	7.46 ± 0.02 (7.42–7.50)^b^	7.46 ± 0.03 (7.40–7.50)^b^	7.47 ± 0.02 (7.41–7.51)^b^	7.47 ± 0.03 (7.42–7.51)^b^	7.38 ± 0.03 (7.35–7.46)^a^	> 7.31; 7.37; 7.42–7.45
tHb [ g/dL ]	11.20 ± 0.87 (9.3–10.3)^b^	10.90 ± 0.71 (9.5–12.5)^b^	11.10 ± 0.92 (9.4–13.1)^b^	11.80 ± 1.21 (8.6–13.1)^b^	9.85 ± 0.63 (8.7–10.7)^a^	8.7–12.4;
HCT [%]	33 ± 2.59 (27–39)^b^	32 ± 2.12 (28–37)^b^	33 ± 2.66 (28–39)^b^	35 ± 3.61 (25–39)^b^	29 ± 1.66^a^ (26–31)	25–33
pCO_2_ [mmHg]	42.10 ± 2.24 (38.6–48.4)^b^	41.90 ± 3.05 (36.1–46.7)^b^	41.60 ± 3.48 (35.4–48.6)^b^	41.25 ± 4.11 (35.0–47.2)^b^	49.70 ± 6.74 (30.9–54.3)^a^	40.43 ± 1.44
pO_2_ [mmHg]	39.05 ± 3.72 (32.8–48.8)^b^	39.10 ± 5.51(22.6–46.5)^b^	35.90 ± 3.99 (29.0–48.5)^b^	40.60 ± 3.81 (32.6–47.1)^b^	27.60 ± 6.54 (24.8–42.3)^a^	33.57 ± 2.11
HCO_3_ − act [mmol/L]	29.75 ± 1.73 (25.90–33.30)^a^	29.10 ± 1.74 (26.0–31.9)^a^	29.50 ± 2.23 (25.7–33.3)^a^	29.80 ± 2.36 (25.7–33.5)^a^	28.95 ± 3.23 (21.3–33.0)^a^	–
HCO_3_ − std [mmol/L]	29.00 ± 1.60 (25.3–32.0)^b^	28.60 ± 1.68 (25.5–30.9)^b^	28.90 ± 1.87 (25.5–32.2)^b^	28.75 ± 1.88 (26.1–32.7)^b^	26.75 ± 2.21 (22.3–30.3)^a^	23.5–27.0
BE(B) [mmol/L]	5.50 ± 1.69 (1.4–8.6)^a^	4.80 ± 1.74 (1.2–7.3)^a^	5.40 ± 2.02 (1.7–8.9)^a^	5.25 ± 2.08 (1.7–9.2)^a^	4.10 ± 2.14 (− 0.4–7.3)^a^	2.51 ± 2.44; − 0.5–4.5
BE ecf [mmol/L]	5.95 ± 1.91 (1.5–9.6)^a^	5.30 ± 1.92 (1.4–8.1)^a^	6.00 ± 2.33 (1.8–9.8)^a^	5.85 ± 2.38 (1.7–10.1)^a^	4.00 ± 3.08 (− 2.5–8.4)^a^	–
ctCO_2_ [mmol/L]	31.05 ± 1.78 (27.2–34.7)^a^	30.40 ± 1.78 (27.1–33.3)^a^	30.80 ± 2.32 (26.8–34.8)	31.10 ± 2.46 (26.8–34.9) ^a^	30.45 ± 3.41 (22.3–34.6)^a^	–
sO_2_ [%]	76.80 ± 5.57 (63.0–86.6)^b^	76.10 ± 10.95 (36.1–86.4)^b^	71.30 ± 7.17 (52.8–85.9)^b^	78.05 ± 6.14 (63.4–83.8)^b^	49.20 ± 14.08 (38.6–78.3)^a^	61.28 ± 1.73
FO_2_Hb [%]	75.70 ± 5.60 (62.1–86.0)^b^	75.40 ± 11.04 (35.5–86.1)^b^	70.30 ± 7.13 (52.1–84.8)^b^	76.95 ± 6.12 (62.4–82.8)^b^	48.50 ± 13.89 (38.0–77.3)^a^	–
FCOHb [%]	0.30 ± 0.22 (0.0–0.8)^a^	0.30 ± 0.33 (0.0–1.2)^a^	0.30 ± 0.30 (0.0–1.3)^a^	0.30 ± 0.15 (0.1–0.6)^a^	0.35 ± 0.16 (0.1–0.6)^a^	–
FMetHb [%]	0.65 ± 0.20 (0.4–1.2)^a^	0.60 ± 0.23 (0.1–1.0)^a^	1.00 ± 0.31 (0.2–1.4)^b^	0.90 ± 0.22 (0.5–1.3)^b^	1.05 ± 0.16 (0.8–1.3)^b^	–
FHHb [%]	22.90 ± 5.48 (13.3–36.4)^a^	23.70 ± 10.70 (13.5–62.8)^a^	28.30 ± 7.05 (13.9–46.5)^b^	21.65 ± 6.05 (16.0–36.1)^b^	50.05 ± 13.87 (21.4–60.5)^b^	–
BO_2_ [mL/dL]	15.50 ± 1.20 (12.7–18.3)^b^	15.00 ± 0.98 (13.0–17.2)^b^	15.30 ± 1.27 (12.9–18.0)^b^	16.25 ± 1.67 (11.8–18.1)^b^	13.45 ± 0.88 (11.9–14.7)^a^	–
p50 [mmHg]	26.35 ± 0.60 (25.0–27.4)^a^	26.80 ± 0.74 (24.9–28.3)^b^	26.90 ± 0.65 (25.8–29.0)^b^	26.65 ± 0.42 (25.8–27.3)^b^	27.60 ± 0.86 (26.2–28.7)^b^	–
ctO2 [mL/dL]	11.75 ± 1.27 (9.3–15.6)^b^	11.50 ± 1.87 (5.5–13.8)^b^	11.40 ± 1.37 (8.2–13.1)^b^	12.00 ± 1.62 (9.8–15.1)^b^	6.55 ± 2.40 (5.1–11.3)^a^	27.24 ± 1.26

The cows were divided in to five groups: I—44 kg milk/cow; II—38 kg milk/cow; III—30 kg milk/cow; IV—25 kg milk/cow; and V—19 kg milk/cow, based on mean daily milk yield. Means with different letters (a–b) are different (*P* < 0.05).

^a–^^c^groups marked by letters differ significantly, *p* ≤ 0.05.

*tHb* Total hemoglobin concentration, *HCT* Hematocrit, *pCO*_*2*_ Partial pressure of carbon dioxide, *pO*_*2*_ Partial pressure of oxygen, *HCO*_*3*_*−act* Actual bicarbonate concentration, *HCO*_*3*_*−std* Standard bicarbonate concentration, *BE(B)* base excess, *BE ecf* Base excess of extracellular fluid, *ctCO*_*2*_ Total carbon dioxide serum concentration, *sO*_*2*_ saturation, *FO2Hb* Oxyhemoglobin, *FCOHb* Carboxyhemoglobin, *FMetHb* Methemoglobin, *FHHb* Deoxyhemoglobin, *BO*_*2*_ Blood oxygen, *p50* Hemoglobin-oxygen affinity, *ctO*_*2*_ Total oxygen concentration.

**Table 4:** Blood ion concentration (median, SD, minimal, and maximal values) in different groups of dairy cattle compared with the normal values for cattle (according to Sayers et al. 2016^30^, Wood et al. 2010^40^, Temiz et al. 2014^37^, and Cornell Animal Health Diagnostic Center^42^).

Parameter	Group I (n = 36)	Group II (n = 21)	Group III (n = 27)	Group IV (n = 18)	Group V (n = 10)	Normal values for bovine species
Na + [mmol/L]	138.00 ± 1.46 (134.6–140.7)^a^	139.10 ± 1.41 (136.1–141.2)^a^	138.30 ± 1.41 (136.0–140.8)^a^	137.95 ± 1.08 (135.1–139.2)^a^	138.85 ± 14.34 (137.2–184.1)^a^	134–144
K + [mmol/L]	4.21 ± 0.26 (3.79–5.11)^a^	4.26 ± 0.36 (3.73–5.35)^a^	4.16 ± 0.21 (3.71–4.64)^a^	4.24 ± 0.28 (3.89–4.71)^a^	4.38 ± 0.39 (4.10–5.48)^a^	4.0–5.9
Ca + + [mmol/L]	1.11 ± 0.04 (1.05–1.20)^a^	1.10 ± 0.06 (0.96–1.21)^a^	1.11 ± 0.05 (0.99–1.16)^a^	1.12 ± 0.04 (1.06–1.21)^a^	1.13 ± 0.13 (0.81–1.32)^a^	1.03–1.32
Ca + + (7.4) [mmol/L]	1.14 ± 0.04 (1.07–1.25)^a^	1.14 ± 0.06 (0.96–1.22)^a^	1.13 ± 0.04 (1.03–1.19)^a^	1.16 ± 0.04 (1.08–1.23)^a^	1.12 ± 0.14 (0.80–1.35)^a^	–
Cl − [mmol/L]	98.0 ± 1.61 (95–102)^a^	99.0 ± 2.64 (95–105)^a^	99.0 ± 1.52 (95–102)^a^	98.0 ± 1.65 (95–102)^a^	98.5 ± 8.81 (96–126)^a^	92–99
AnGap [mmol/L]	14.45 ± 1.30 (11.7–16.7)^a^	15.00 ± 1.80 (10.9–17.7)^a^	14.20 ± 1.92 (10.8–18.2)^a^	14.15 ± 1.69 (10.4–16.2)^a^	15.15 ± 8.85 (12.0–42.2)^a^	19–26
Glu [mg/dL]	58 ± 5.50 (47–72)^a^	52 ± 8.63 (40–75)^a^	55 ± 5.74 (50–73)^a^	62 ± 7.54 (49–76)^b^	67 ± 5.20 (56–72)^b^	57–79

The cows were divided in to five groups: I—44 kg milk/cow; II—38 kg milk/cow; III—30 kg milk/cow; IV—25 kg milk/cow; and V—19 kg milk/cow, based on mean daily milk yield. Means with different letters (a–b) are different (*P* < 0.05).

^a–^^b^means with different letters are different (*P* < 0.05).

*AnGap *Anion gap, *Glu* Glucose concentration.

**Figure 2:**
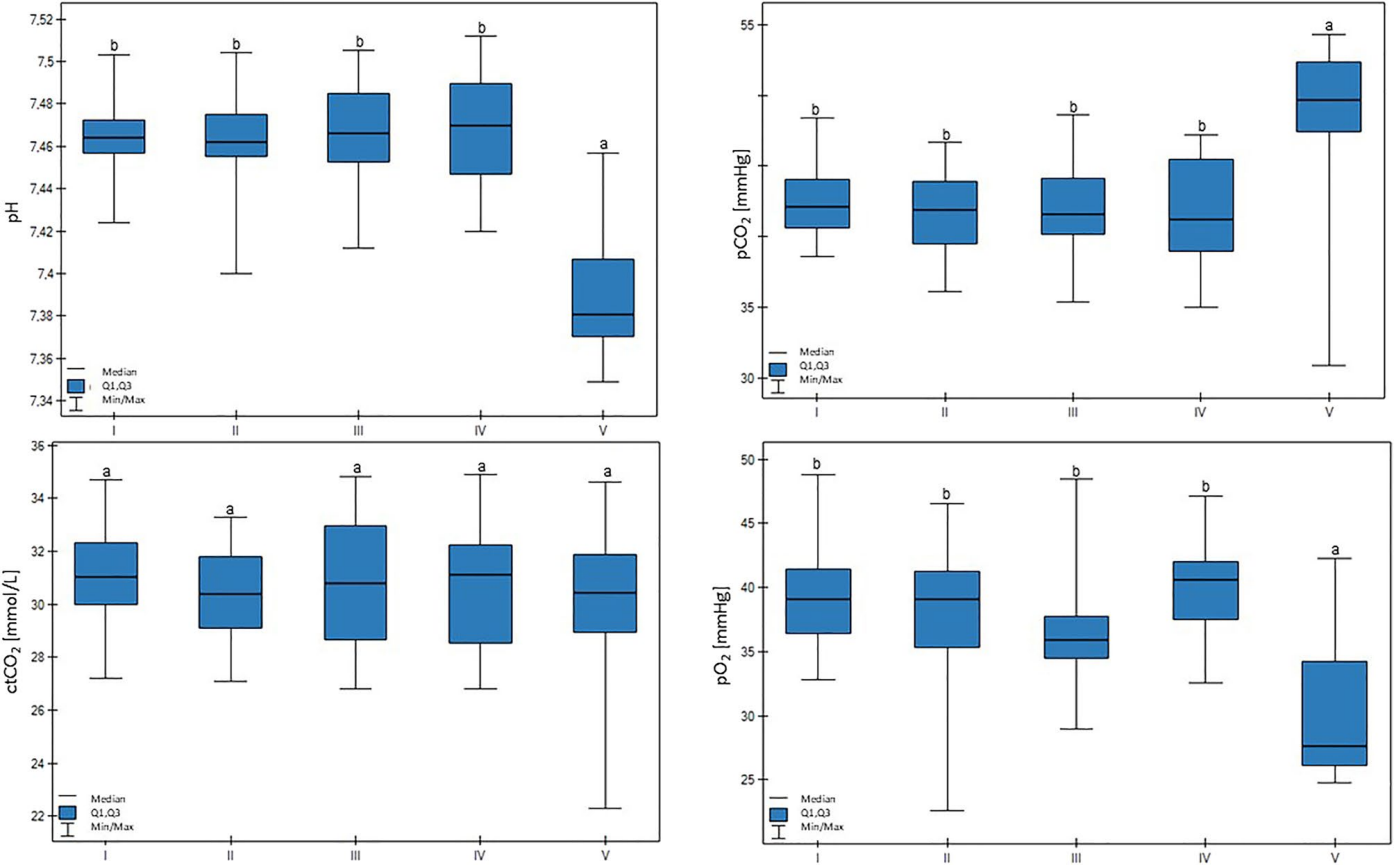
Differences in pH, pO_2_ (partial pressure of oxygen), pCO_2_ (partial pressure of carbon dioxide), and ctCO_2_ (total carbon dioxide concentration) values in cows at different production levels. In the box plots, the upper whisker represents the maximum value; the upper line of the box represents Q3 (upper quartile); the center line inside the box represents the median; the lower line of the box represents Q1 (lower quartile); and the lower whisker represents the minimum value. Groups marked by letters (a–b) differ significantly, p ≤ 0.05. The cows were divided in to five groups: I—44–39 kg milk/cow; II – 38–31 kg milk/cow; III—30–26 kg milk/cow; IV—25–20 kg milk/cow; and V—19–17 kg milk/cow, based on mean daily milk yield.

A lower pH value was measured in group V (*p* < 0.01). Group V also had lower pO_2_ compared to the other groups (*p* < 0.05). In this case, a significant trend was found: the lower the milk yield, the lower the pO_2_ value (*p* < 0.05). The pCO_2_ value was higher for group V (*p* < 0.01). Group V was characterized by lower ctO2 values (*p* < 0.01), and decreased milk production was related to decreased ctO_2_ (*p* < 0.05). These results are shown in Fig. 2.

**Figure 3:**
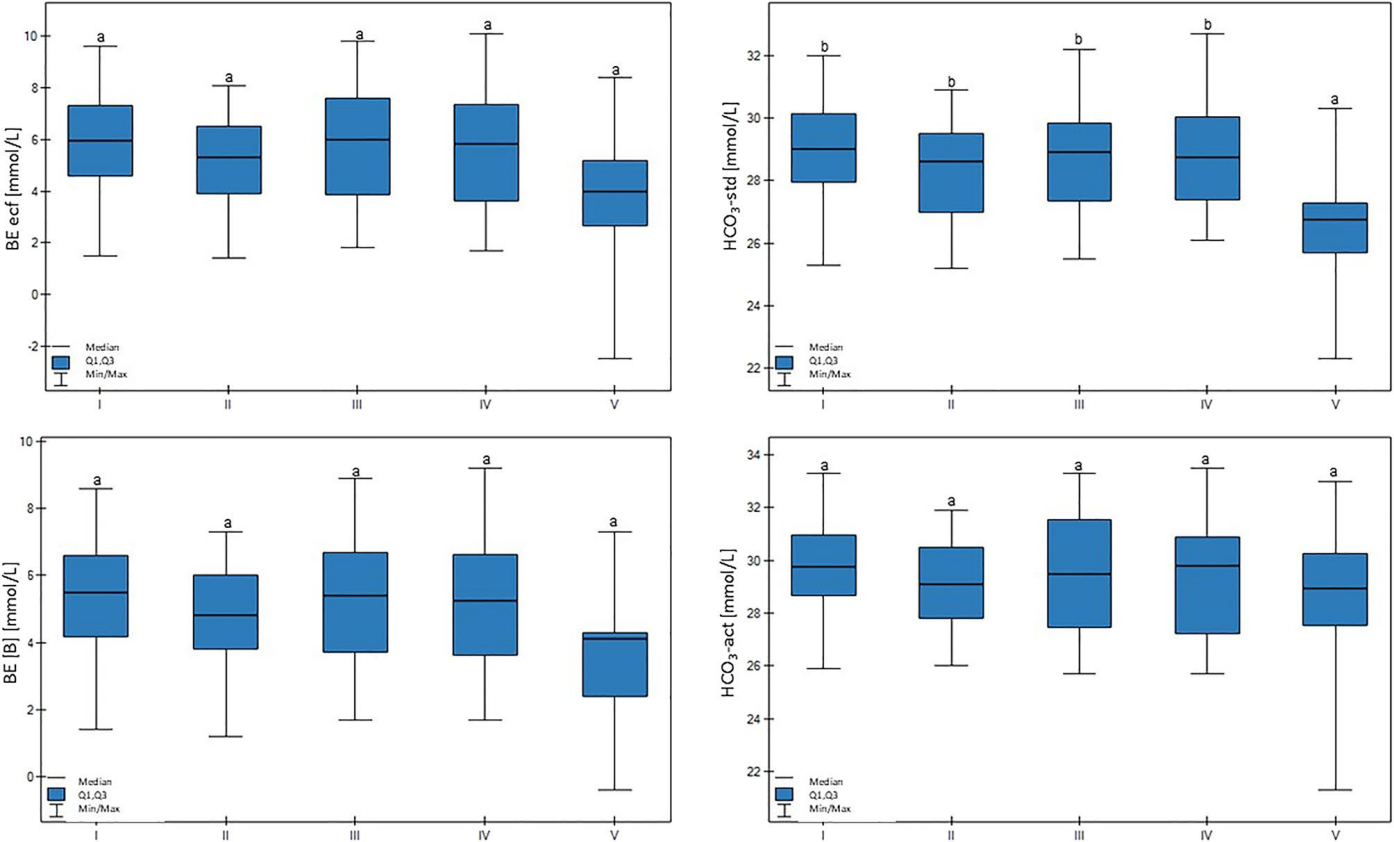
Differences in BE(B) (base excess), BE ecf (base excess of extracellular fluid), HCO_3_ − act (actual bicarbonate concentration), and HCO_3_ − std (standard bicarbonate concentration) values in cows at different production levels. In the box plots, the upper whisker represents the maximum value; the upper line of the box represents Q3 (upper quartile); the center line inside the box represents the median; the lower line of the box represents Q1 (lower quartile); and the lower whisker represents the minimum value. Groups marked by letters (a–b) differ significantly, p ≤ 0.05. The cows were divided in to five groups: I—44–39 kg milk/cow; II—38–31 kg milk/cow; III—30–26 kg milk/cow; IV—25–20 kg milk/cow; and V—19–17 kg milk/cow, based on mean daily milk yield.

The HCO_3_−std value was lower in group V in comparison to other groups (*p* > 0.01). Here, a another significant trend was confirmed: lower milk yield was associated with lower HCO_3_−std values (*p* < 0.05). There were no differences in the HCO_3_−act, BE(B), or BE ecf values between the groups. These results are shown in Fig. 3.

**Figure 4:**
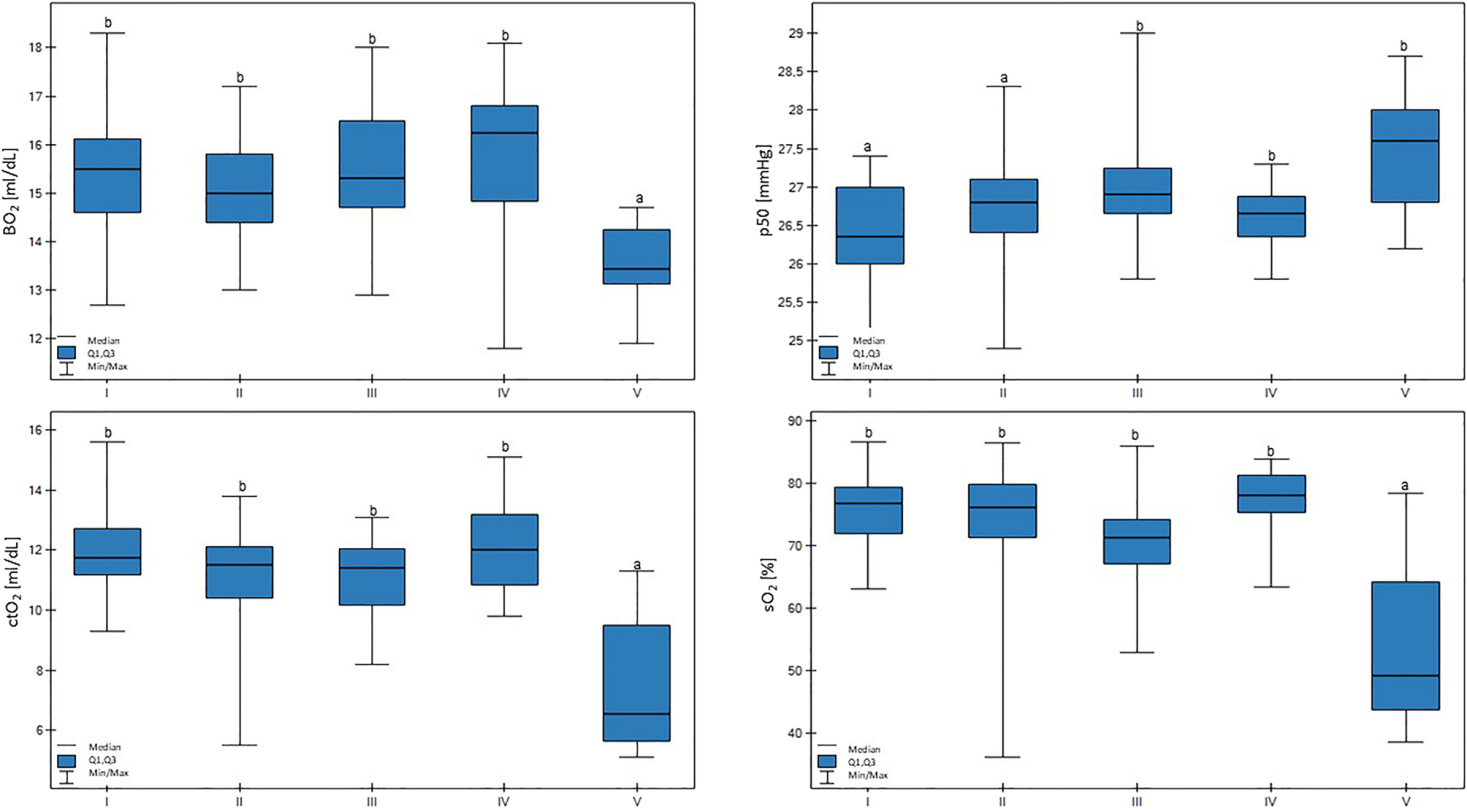
Differences in BO_2_ (blood oxygen), p50 (hemoglobin-oxygen affinity), ctO_2_ (total oxygen concentration), and sO_2_ (saturation) values in cows at different production levels. In the box plots, the upper whisker represents the maximum value; the upper line of the box represents Q3 (upper quartile); the center line inside the box represents the median; the lower line of the box represents Q1 (lower quartile); and the lower whisker represents the minimum value. Groups marked by letters (a–b) differ significantly, p ≤ 0.05. The cows were divided in to five groups: I – 44—39 kg milk/cow; II – 38 -31 kg milk/cow; III – 30–26 kg milk/cow; IV – 25–20 kg milk/cow; and V – 19–17 kg milk/cow based on mean daily milk yield.

In group V, the sO_2_ values were lower than all the other groups (*p* < 0.01). In addition, a trend for lower milk yield with lower sO_2_ values was confirmed (*p* < 0.01). In group V, the BO_2_ value was lower than in the other groups (*p* < 0.01). Low p50 values were connected with lower milk productivity (*p* < 0.01), and, additionally, the lowest p50 values were found in group I, which were lower than groups III, IV, and V (*p* < 0.01). There were no differences in the ctO2 values. These results are shown in Fig. 4.

**Figure 5:**
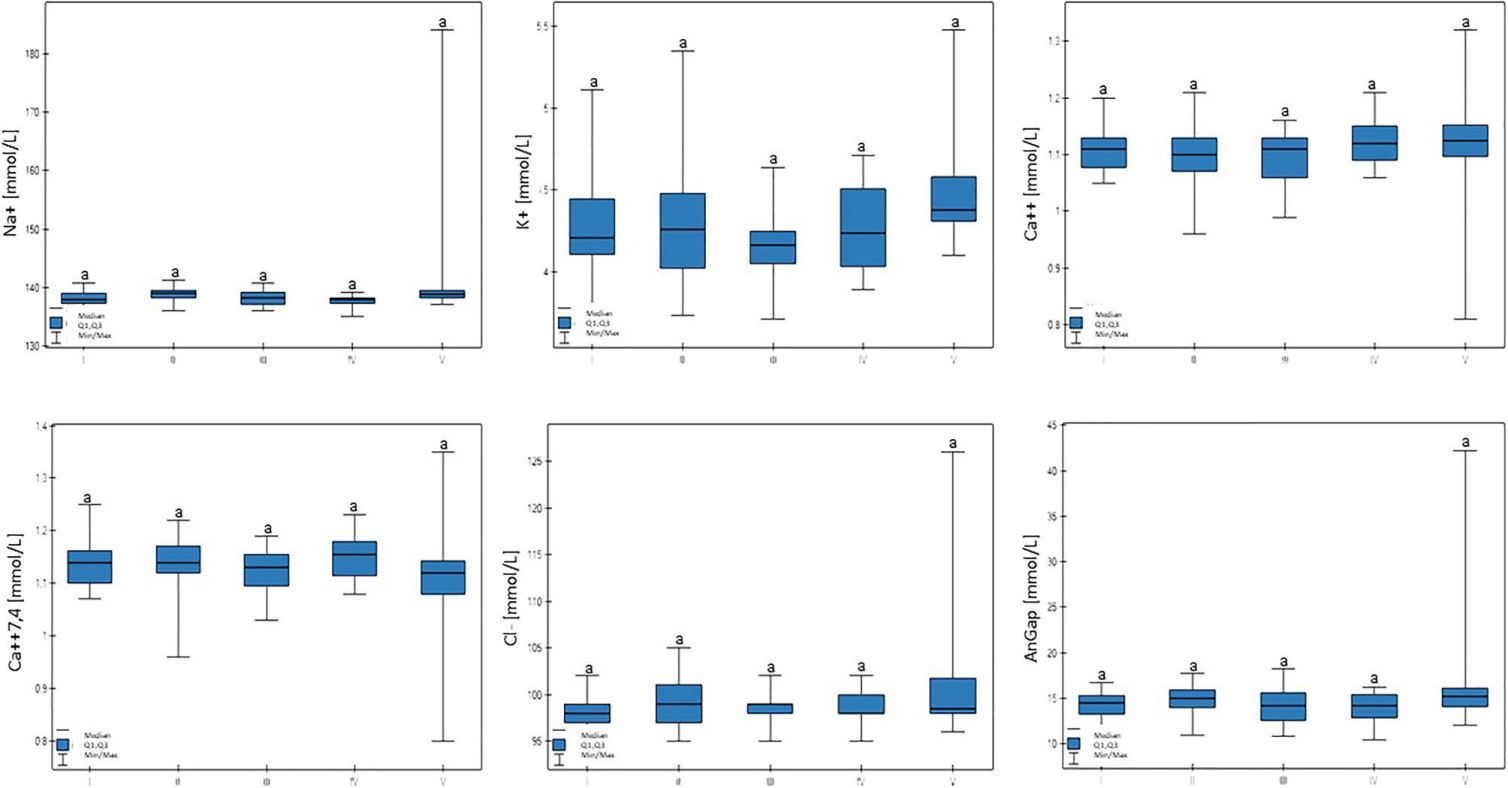
Differences in Na + , K + , Ca +  + , Ca +  + (7,4), Cl − , and AnGap (anion gap) values in cows at different production levels. In the box plots, the upper whisker represents the maximum value; the upper line of the box represents Q3 (upper quartile); the center line inside the box represents the median; the lower line of the box represents Q1 (lower quartile); and the lower whisker represents the minimum value. Groups marked by letters (a–b) differ significantly, p ≤ 0.05. The cows were divided in to five groups: I—44–39 kg milk/cow; II—38–31 kg milk/cow; III—30–26 kg milk/cow; IV—25–20 kg milk/cow; and V—19–17 kg milk/cow, based on mean daily milk yield.

There were no statistically significant differences between Na+, K+, Ca++, Ca++(7.4), or Cl−, or the AnGap and glucose serum concentrations (Table 4, Fig. 5).

**Figure 6:**
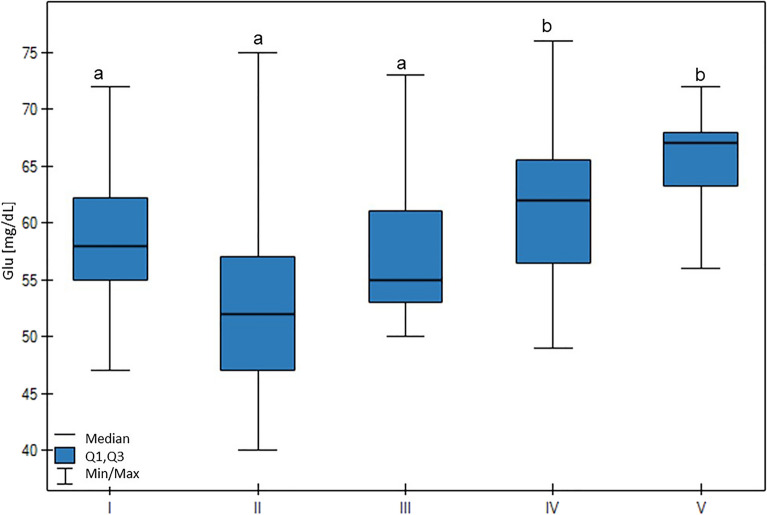
Differences in Glu (glucose) values in cows at different production levels. In the box plots, the upper whisker represents the maximum value; the upper line of the box represents Q3 (upper quartile); the center line inside the box represents the median; the lower line of the box represents Q1 (lower quartile); and the lower whisker represents the minimum value. Groups marked by letters (a–b) differ significantly, p ≤ 0.05. The cows were divided in to five groups: I—44–39 kg milk/cow; II—38–31 kg milk/cow; III—30–26 kg milk/cow; IV—25–20 kg milk/cow; and V—19–17 kg milk/cow, based on mean daily milk yield.

A high Glu blood concentration was associated with low milk production (*p* < 0.01), with the highest Glu concentration being found in group V, which was higher than groups I, II, and III (*p* < 0.01) (Fig. 6).

## Discussion

To the authors’ best knowledge, there is little up to date data regarding the reference values for blood gas analysis for dairy cattle producing different milk yields^19,20^. In a recent study, reference values were established in calves for parameters commonly used in human blood gas analysis, such as standard HCO_3_− ; actual HCO_3_− ; pCO_2;_ the ions Na+ , K+ , Ca++, and Cl−; glucose; tHb; and AnGap^27^. However, there is a lack of studies concerning the basal gas blood parameters for adult dairy cattle at different milk production levels. Parameters such as BE ecf, FO_2_Hb, FCOHb, FMetHb, FHHb, BO_2_, p50, ctO_2_, and ctCO_2_, have never been reported together in bovine species. The results of our study indicate that differences in milk production levels appear to correlate with changes in the blood gas profiles of HF cattle. Therefore, production levels may need to be taken into consideration when establishing reference intervals for dairy cattle.

### Hematocrit, hemoglobin products, and hemoglobin-oxygen affinity parameters

Ht and tHb values were lower in cattle that produced 18–20 kg of milk/cow. In addition there were differences in the concentration of the Hb products^28^. FO_2_Hb (oxyhemoglobin) defines the amount of oxygen-carrying hemoglobin in the blood. There was a trend confirming that a decrease in FO_2_Hb concentration was connected with lower milk yield, as well as with a high percentage of deoxyhemoglobin (FHHb)—the form of Hb in the blood without oxygen—and methemoglobin (FMetHb) content in the blood. Increased levels of FMetHb confirmed the progression of various types of oxidative damage in erythrocytes, and caused a leftward shift in the hemoglobin dissociation curve^29^. The differences were most visible in cows producing more than 38 kg of milk (group I and II), in which the FMetHb value was almost two times lower (0.6% vs. 1.05%) than in other groups. Taken all together, the changes in the concentration of Hb products in the blood influenced the Hb’s affinity for oxygen as well as the release of oxygen^28^. This may be connected with lower oxygen demand by the tissue that produces the lower amounts of milk. The decreased partial pressure of oxygen in the blood (left shift) indicated the hemoglobin’s increased oxygen affinity, allowing less oxygen to be available to the tissues. Low oxygen partial pressures decrease metabolic rates through several cellular tolerance strategies by activating anaerobic energy production; however, this is less effective. In addition, a lower hemoglobin-oxygen affinity was also confirmed by the increased P50 values and decreased sO_2_, BO_2_, and ctO_2_ values in cows producing 18–20 kg of milk. It has been confirmed that increased milk production enhances blood supply to the mammary gland in dairy cattle, and so oxygen consumption is correlated with blood flow^30,31^. However, the tissue’s nutritional and oxygen demands seems to be stable, whereas the blood flow may change^32^. It has been postulated that mammary blood flow is mainly a function of the number of lactations, therefore, we tried to select cows of similar age, so there were minimal differences between the number of lactations^31^. This study confirms that the blood oxygen concentration, and the ability to transport it, is increased in cattle producing higher amounts of milk. However, the levels for the mentioned parameters were still physiological. Little information is available concerning the meaning of the changes to the levels of Hb products in cattle, and it has not yet been established that manipulating these levels during illness can improve gas exchange, tissue oxygenation, or outcome. However, the differences between normal and high-producing herds should be taken into account when performing clinical examinations of the blood of such animals.

### The pH and buffer systems

It has been suggested that monitoring blood pH alone is useful because of the strong correlation between pH and clinical health; however, additional parameters are important because they may lead to a conclusion as to whether the problem is primarily metabolic or respiratory^15,33^. Changes in blood oxygen level influence compensatory mechanisms and causes the rapid disintegration of O2 and CO2 carrying red blood cells (RBC) in animals.

The simultaneous quick release of the RBCs’ contents seems to cause changes in blood pH^34^. In cows producing 18–20 kg of milk, the blood pH was lower (7.38) than the other groups (7.46–7.47); however, the values were still higher than the established lower reference value for bovines (< 7.31)^15^. This was accompanied by higher pCO_2_ and lower pO_2_ levels_._ In addition, HCO_3_− std was slightly decreased in these animals. It is well known that pCO_2_ is correlated with HCO_3_− values because the majority of carbon dioxide (CO_2_) is carried within this buffer system^33^. However, in most cases, as in our study, the HCO_3_− values are calculated automatically by the analyzer from pCO_2_ levels.

Beyond the RBC parameters, other factors should also be considered as reasons why the pH is decreased in low producing milk cows. The calculated anion gap reflects the amount of immeasurable ions, which are primarily anions, hence the name "anion gap." However, in our study this parameter remained unchanged across all groups. Ketoacidosis may cause high anion gap metabolic acidosis, whose affects are more common in high-production farms; in many cases it is subclinical. In our study, the ketone level in blood was not measured, nor was lactate blood level, which may also influence pH^35^. Chronic rumen acidosis may lead to lactic acidosis and a decrease in blood pH because there is a strong relationship between the rumen pH and blood pH^36^. However, it is impossible to exclude all subclinical disorders.

### Ions

The systemic generation of HCO_3_− is also connected with the evolution of ions. Ions such as Na+ , K+ , Ca++ , and Cl^−^ play a major role in determining serum electrolytes^37^. They influence the osmotic balance, acid base integrity, the pumping mechanisms of cell membranes, and the energy production requirements for the synthesis of milk ingredients such as lactose^38^. During increased lactogenesis, high-producing dairy cows undergo extreme metabolic adaptations that are critical in defining the success of the oncoming lactation. Mineral requirements as well as energy and protein needs are increased when multiple organs orchestrate these metabolic adaptations^39^. It has been postulated that blood ion concentration changes due to increased milk production are caused by free water movement into the udder^37^. However this phenomenon has been described mostly just after calving, as the most dramatic changes in cell metabolism occur in order to support enhanced milk production during this period^40^. The transition from pregnancy to lactation imposes enormous stress on dairy cows, greatly increasing their susceptibility to metabolic disorders. In our study the largest range in serum ions occurred in the lowest producing group. This may be connected with subclinical disorders that may never have been excluded, and that may influence the lower production rate in this group.

### Glucose

Glucose provides energy for maintenance and production. In this study, glucose blood concentrations were lower in cattle producing more than 24 kg milk per cow (52–62 vs 67 mg/dL). Thus, lower blood glucose levels are induced by the high rates of utilization needed to fulfill the requirements of the mammary gland for higher milk synthesis^41^. This is in line with other findings—that lower blood glucose levels may be related to increased requirements for milk production^42^.

### Limitations

The main limitation in our study was the number of cows in the examined groups, which were too small to establish reference values according to the American Society for Veterinary Clinical Pathology’s guidelines for the determination of reference intervals in veterinary species^43^. Thus, there is need for further analysis using a larger data set, which would be beneficial in defining suitable cut-off values, thus contributing to the thresholds and scope of blood gas ranges for different milk production level dairy cattle. In addition, the test methodology and laboratory equipment may have influenced the differences obtained for the parameters. It should be mentioned that changes in parameters such as pH and HCO_3_− , and excess base and ion concentrations in the blood, may be disrupted by the dietary cation-anion difference (DCAD)^36^. Most nutritional supplements are more or less similar in content, however, to minimize environmental influence, only animals from one herd were selected for the study. In addition, it has been postulated that not all nutrients affect blood parameters^44^. Other limitation may be connected with sample handling, because it is advisable that blood gas analysis be performed within 15 min of blood collection. However, there are publications that indicate that when blood is stored at 4 °C, changes may be delayed^45^. In connection with the low biologic variability of some blood gas parameters, it has been stated that very little error resulting from specimen collection can be tolerated^46^. In clinical practice connected with food producing animals, it is very often impossible to perform hematological examination within five minutes because the blood samples have to be transported to a laboratory, in contrast to procedures for equine or small animal practice.

## Conclusions

In conclusion; our study demonstrated subtle differences in the hematologic values for HF cows with different levels of milk production. While comparisons cannot be made directly, the results provide evidence that milk production may affect blood gas parameters as well as pH levels, which should be considered when evaluating clinically ill cattle. These days, the ready availability of simplified, economical, and portable diagnostic equipment may, therefore, improve early, accurate diagnoses and prognoses. Changes in blood gas parameters may be helpful in the early-stage diagnosis, monitoring, and prognosis of a disease.

## Data Availability

The datasets used and/or analyzed during the current study are available from the corresponding author on reasonable request.

## Abbreviations

AnGap: Anion gap
BCS: BODY condition score
BE ecf: Base excess of extracellular fluid
BE(B): Base excess
BO_2_: Blood oxygen
ctCO_2_: Total carbon dioxide serum concentration
ctO_2_: Total oxygen concentration
DCAD: Dietary cation-anion difference
EU: European Union
FCOHb: Carboxyhemoglobin
FHHb: Deoxyhemoglobin
FMetHb: Methemoglobin
FO_2_Hb: Oxyhemoglobin
Glu: Glucose
HCO_3_−act: Actual bicarbonate concentration
HCO_3_−std: Standard bicarbonate concentration
HF: Holstein-Friesian
Ht: Hematocrit
IQR: Interquartile range
p50: Hemoglobin-oxygen affinity
pCO_2_: Partial pressure of carbon dioxide
pO_2_: Partial pressure of oxygen
RBC: Red blood cells
SD: Standard deviation
sO_2_: Saturation
tHb: Total hemoglobin concentration
TMR: Total mixed ration
WULS: Warsaw University of life sciences

## References

[1] The Embassy of the Netherlands in Poland. Quick Scan Polish Dairy Sector Contents : [Internet]. Available from: https://www.agroberichtenbuitenland.nl/actueel/nieuws/2020/10/21/quick-scan-polish-dairy-sector.

[2] Sznajder M, Krusińska O, Wielicka A (2005). Review of dairy sector in Poland. J. Agribus. Rural Dev..

[3] Haque N, Patel A, Lateef A, Patel P, Bhalakiya N (2017). Study on blood metabolites and leukocyte indices of kutchi camels during different stages of lactation. J. Anim. Health Prod..

[4] Brotherstone S, Goddard M (2005). Artificial selection and maintenance of genetic variance in the global dairy cow population. Philos. Trans. R. Soc. Lond. B Biol. Sci..

[5] Meyerholz MM, Rohmeier L, Eickhoff T, Hülsebusch A, Jander S, Linden M, Macias L, Koy M, Heimes A, Gorríz-Martín L, Segelke D, Engelmann S, Schmicke M, Hoedemaker M, Petzl W, Zerbe H, Schuberth HJ, Kühn C (2019). Genetic selection for bovine chromosome 18 haplotypes associated with divergent somatic cell score affects postpartum reproductive and metabolic performance. J. Dairy Sci..

[6] Roland L, Drillich M, Iwersen M (2014). Hematology as a diagnostic tool in bovine medicine. J. Vet. Diagn. Investig..

[7] Coroian CO, Miresan V, Coroian A, Raducu C, Andronie L, Marchis Z (2017). Biochemical and haematological blood parameters at different stages of lactation in cows. Bull. Univ. Agric. Sci. Vet. Med. Cluj-Napoca Anim. Sci. Biotechnol..

[8] Ruginosu E, Creangă S, Sofronie M, Mălăncuş R, Boghian V, Solcan G (2011). The biochemical profile in cows with reproductive disorders. Cercet. Agron. în Mold..

[9] Radostits OM (2003). Engineering veterinary education: A clarion call for reform in veterinary education-let’s do it!. J. Vet. Med. Educ..

[10] Ha S, Kang S, Han M, Lee J, Chung H, Oh S-I (2022). Predicting ketosis during the transition period in Holstein Friesian cows using hematological and serum biochemical parameters on the calving date. Sci. Rep..

[11] Radkowska I, Herbut E (2014). Hematological and biochemical blood parameters in dairy cows depending on the management system. Anim. Sci. Pap. Rep..

[12] Mazzullo G, Rifici C, Caccamo G, Rizzo M, Piccione G (2014). Effect of different environmental conditions on some haematological parameters in cow. Ann. Anim. Sci..

[13] Cozzi G, Ravarotto L, Gottardo F, Stefani AL, Contiero B, Moro L (2011). Short communication: Reference values for blood parameters in Holstein dairy cows: Effects of parity, stage of lactation, and season of production. J.Dairy Sci..

[14] Imhasly S, Naegeli H, Baumann S, von Bergen M, Luch A, Jungnickel H (2014). Metabolomic biomarkers correlating with hepatic lipidosis in dairy cows. BMC Vet. Res..

[15] Sayers RG, Kennedy A, Krump L, Sayers GP, Kennedy E (2016). An observational study using blood gas analysis to assess neonatal calf diarrhea and subsequent recovery with a European Commission-compliant oral electrolyte solution. J. Dairy Sci..

[16] Mohri M, Sharifi K, Eidi S (2007). Hematology and serum biochemistry of Holstein dairy calves: Age related changes and comparison with blood composition in adults. Res. Vet. Sci..

[17] Wood, D. & Quiroz-Rocha, G. F. Normal hematology of cattle. *Schalm's Vet. Hematol.* 829–35 (2010).

[18] Divers TJ, Peek SF (2008). Rebhun’s Diseases of Dairy Cattle.

[19] Braun U, Forster E (2012). B-mode and colour Doppler sonographic examination of the milk vein and musculophrenic vein in dry cows and cows with a milk yield of 10 and 20 kg. Acta Vet. Scand..

[20] Bovo S, Mazzoni G, Bertolini F, Schiavo G, Galimberti G, Gallo M, Dall'Olio S, Fontanesi L (2019). Genome-wide association studies for 30 haematological and blood clinical-biochemical traits in Large White pigs reveal genomic regions affecting intermediate phenotypes. Sci. Rep..

[21] Comline RS, Silver M (1974). A comparative study of blood gas tensions, oxygen affinity and red cell 2,3 DPG concentrations in foetal and maternal blood in the mare, cow and sow. J. Physiol..

[22] Szenci O, Besser T (1990). Changes in blood gas and acid-base values of bovine venous blood during storage. J. Am. Vet. Med. Assoc..

[23] Naito Y, Murakami D (1982). Blood gas and acid-base values in the coccygeal artery of holstein-friesian cows. Jpn. J. Vet. Sci..

[24] Piccione G, Caola G, Mortola JP (2004). Day/night pattern of arterial blood gases in the cow. Respir. Physiol. Neurobiol..

[25] Millius L, Riedo E, Caron T, Belissent J, Fellay B, Ribordy V, Magnin JL (2021). The "EPiQ"-Study (Evaluation of preanalytical quality): S-Monovette® in manual aspiration mode drastically reduces hemolytic samples in head-to-head study. Pract. Lab. Med..

[26] National Committee for Clinical Laboratory Standards (NCCLS) Document C12–T2 (1991). Definitions of Quantities and Conventions Related to Blood pH and Gas Analysis.

[27] Dillane P, Krump L, Kennedy A, Sayers RG, Sayers GP (2018). Establishing blood gas ranges in healthy bovine neonates differentiated by age, sex, and breed type. J. Dairy Sci..

[28] Patel, S., Jose, A., Mohiuddin, S. S. Physiology, oxygen transport and carbon dioxide dissociation curve. StatPearls. 2022. Available from: http://www.ncbi.nlm.nih.gov/pubmed/30969637. 30969637

[29] Shiono H, Yagi Y, Thongnoon P, Kurabayashi N, Chikayama Y, Miyazaki S (2001). Acquired methemoglobinemia in anemic cattle infected with Theileria sergenti. Vet. Parasitol..

[30] Berger H, Lietzau M, Tichy A, Herzog K (2016). Investigations of mammary and uterine blood flow in relation to milk yield, postpartum disease, and pregnancy result in dairy cows. Theriogenology.

[31] Götze A, Honnens A, Flachowsky G, Bollwein H (2010). Variability of mammary blood flow in lactating Holstein-Friesian cows during the first twelve weeks of lactation. J. Dairy Sci..

[32] Hue-Beauvais C, Faulconnier Y, Charlier M, Leroux C (2021). Nutritional regulation of mammary gland development and milk synthesis in animal models and dairy species. Genes (Basel).

[33] Burns GP (2014). Arterial blood gases made easy. Clin. Med. (Northfield Il).

[34] Temiz M, Altuğ N, Yüksek N (2014). Relationship between degree of anemia and blood gases in cattle with theileriosis. Turkish J. Vet. Anim. Sci..

[35] Yildiz R, Aydogdu U, Guzelbektes H, Coskun A, Sen I (2017). Venous lactate, pH and partial pressure of carbon dioxide levels as prognostic indicators in 110 premature calves with respiratory distress syndrome. Vet. Rec..

[36] Gianesella M, Morgante M, Cannizzo C, Stefani A, Dalvit P, Messina V, Giudice E (2010). Subacute ruminal acidosis and evaluation of blood gas analysis in dairy cow. Vet. Med. Int..

[37] Muiño R, Hernández J, Benedito JL, Castillo C (2021). Effects of calving body condition score on blood acid-base balance of primiparous holstein-friesian dairy cows in a commercial dairy farm: A case study. Animals.

[38] Zebeli Q, Mansmann D, Steingass H, Ametaj BN (2010). Balancing diets for physically effective fibre and ruminally degradable starch: A key to lower the risk of sub-acute rumen acidosis and improve productivity of dairy cattle. Livest Sci..

[39] Roche JR, Bell AW, Overton TR, Loor JJ (2013). Nutritional management of the transition cow in the 21st century—a paradigm shift in thinking. Anim. Prod. Sci..

[40] Hu W, Murphy MR (2004). Dietary cation-anion difference effects on performance and acid-base status of lactating dairy cows: A meta-analysis. J. Dairy Sci..

[41] Omari M, Lange A, Plöntzke J, Röblitz S (2020). Model-based exploration of the impact of glucose metabolism on the estrous cycle dynamics in dairy cows. Biol. Direct.

[42] García-Roche M, Cañibe G, Casal A, Mattiauda DA, Ceriani M, Jasinsky A (2021). Glucose and fatty acid metabolism of dairy cows in a total mixed ration or pasture-based system during lactation. Front. Anim. Sci..

[43] Friedrichs KR, Harr KE, Freeman KP, Szladovits B, Walton RM, Barnhart KF (2012). ASVCP reference interval guidelines: Determination of de novo reference intervals in veterinary species and other related topics. Vet. Clin. Pathol..

[44] Owens FN, Secrist DS, Hill WJ, Gill DR (1998). Acidosis in cattle: A review. J. Anim. Sci..

[45] Picandet V, Jeanneret S, Lavoie JP (2007). Effects of syringe type and storage temperature on results of blood gas analysis in arterial blood of horses. J. Vet. Intern. Med..

[46] Harding PJ, Fraser CG (1987). Biological variation of blood acid-base status: Consequences for analytical goal-setting and interpretation of results. Clin. Chem..

